# 

**Published:** 1830

**Authors:** 


32. *To subscribers, and the profession generally.*—The Editor of this Journal has disposed of the work to Dr. James C. Finley, who will hereafter be an associate Editor, and has engaged as publishers Messrs. Robbins and Deming of this city. Dr. Finley is a gentleman of talents and education, and intends to devote himself to the Journal with great industry and perseverance. His functions will commence with the first number of the fourth volume, now about to be committed to the press. From this arrangement, the work cannot fail to experience a decided improvemment, and become much more worthy of public patronage than it has heretofore been. Its plan will remain unchanged; but more editorial labour will be devoted to its pages, especially in the department of reviews, and its publication will be effected with a degree of punctuality, which the Editor with his numerous engagements has hitherto found impracticable.

The prospectus on the back of this number, will communicate to subscribers and the profession at large, the necessary information concerning this arrangement.

### INDEX TO VOLUME THIRD.

A.

Amaurosis, . . . 122
Amputation, one incision, 122
Ascites, cured by pressure, 123
  case of, . . . 124
Abstinence, effects of, . 131
  emaciation, . . 131
  heart and blood vessels, . 131
  stomach, . . 131
  intestinal tube, . 132
  blood, lymph, mucous membrane, 132
Angina Pectoris, . 146
Aneurism, tying the artery on the distal side, . 301
Aneurism, auticardial operation for, . . 587

B.

Burns and Scalds, . 141
  flour remedy for, . 141
  case, . . 141
  modus curandi, . 142
Brain, watery effusion in, 142
Brain, inflammationof the sinuses of, . . 435
Blood and bloodvessels, state of inflammation, . 261
Blood coagulation of, . 264
Blood of man, peculiar odorous principle in, . 566

C.

Chest, diseases of, . 68
  history, the lungs, . 60
  percussion, . 70
  symptoms and mode of practice, . 70, 71, 72, 73
  auscultation, . 73
  stethoscope, . 73
  description of, . 74
  plate, . . 74
  directions for use, . 76
  phenomena in, . 77
  respiration, . 78
  sonorous rattle, . 80
  sibilant or hissing, . 80
  crepitating, . 81
  voice, phenomena of, . 81
  resonance, . . 81
  pectriloquy, . 82
  ægophany, . . 83
  metallic respiration, . 85
  metallic tinkling, . 85
  practical application, 87
  pleurodynia, . 87
  bronchitis, . . 87
  hœmoptysis, . 88
  oedema of the lungs, 68
  emphysema, . 88
  pneumonia, . 89
  pleurisy, . 91
  pneumo thorax, . 94
  remarks, general, 97, 98, 99
Chiblains, iodine for, . 120
Caustic paste, . . 130
Cornea, opacity of, . 131
Collyrium siccum, . 131
Colica pictonum, . 140
Cutaneous diseases, . 148
  treatment, . 149
Centinarian, death of 154
Cheiloplastic, . 426
Combustion, spontaneous, 427
Camphor, solution of, . 430
Corrosive sublimate, detection of, . 436
Cinchona Bark, new organic alkalies in, . 430
Chimioidia, . 438
Consumption, tubercular, 448
  antiphlogistic treatment injurious effects of, 447
  exercise, effects of, 448
  medicinal, . 450
Cynanche Trachealis, 451
Cancer, Chloride of sodium in . . 453
Consumption, negro case of, 193
  examination, post mortem, 192
Chest, diseases of, . 222
  stethoscope in, . 222
  remarks on the instrument, . . 223, 224
  heart, phenomena natural, 224
  palpitation, . 225
  shock and noise, . 225

rhythm. . 226
morbid phenomena, 227
extent and impulse, 227
the bellows blast, 228
noise of the rasp or file, 229
crackling or leather sound like, and rhythm, 229
general semiology, 231
classification, cardiac maladies, . . 231
malformations and muscular læsions, . 232
degeneracies. . 233
muscular læsions, simple hypertrophy, } 233
morbid anatomy of, 234
dilatations, ventricles, 235
diagnosis, . 235
morbid anatomy of, 236
hypertrophy with dilatation, . 237
left ventricle diagnosis, right ventricle diagnosis } 237
morbid anatomy of each, 228
dilatation of auricles, with hypertrophy, . 238
diagnosis and morbid anatomy, . . 238
aneurisms, . 238
diagnosis of, . 239
degeneracies, . 241
adipose, diagnosis of, 241
bony, . 241
valves of, . 241
diagnosis of, . 241
Coronary arteries, ossification of, . 242
morbid anatomy of, 242
softening, . 242
Cardits and pericardits, 243
morbid anatomy, . 244
hydropericardium, 244
palpitations and spasmodic irregularities, . 246
diagnosis, . 246
irritation from venesection, 246
dyspepsia, . 246
hypochondraism, . 248
chronic hepatitis, . 248
chlorosis, . 249
menorrhagia, and hysteria, 249
grief and apprehension, 250
rheumatism of, . 252
disorders occasioned by, 253
brain, . . 253
lungs, . . 254
liver, . . 255
membrane serous and reticulated, . 256
prognosis and treatment, 257
abstinence and bloodletting, . . 258
cathartics and diuretics, 250
expectorants, . 260
revulsants, narcotics, antispasmodics, and external stimulation, - 260
Cataract and glaucoma, 270
Children, reversed presentation of, . . 299
Calica pictonum postmortem appearances, . 312
Cæsarian operation, successful case of, . 485
state of the patient, . 485
operation, . 486
after treatment, . 488
Colchicum autumnale, effects of, . . 489
Cinchonœ cordifoliœ, liquor of, . . 561
macerations, . 563
papins digester, acid decoction, . . 564
Camphor, effects of, . 575
Circulation, . . 581
Circulation, arterial, action of arteries in, . 585
Choræ, tartar emetic ointment in, . . 590
Consumption, pulmonary, treatment of, } 590

D.

Dysentery, . . 121
Dengue, account of, . 347
symtoms, . . 347
treatment, . 350
Diamonds, artificial, 295
Dengue, . . 304

E.

Education Medical, . 13
pupils, selection and education, . . 13 to 22
learned languages, . 23
age, . . . 25
Empyema, case of, . 65
operation for, . 66
after treatment, . 66, 67
Education, medical, . 318
private pupilage, . 318
choice of preceptors, ,319
time of commencing study 321

age, . . 322

time of study, . 324

collateral studies, 326, 327

plan of conducting, 329

chemistry, . 329

anatomy, . 329

physiology, general, pathology, botany, materia medica, 329

physic and surgery, 332

nosology, . 333

œtiology, practice, 334

obstetrics, . 337

Eye, physiology of, . 355

Eutropion, cure for, 452

Epilepsy, case suspended by a burn, . . 209

Ergot of rye, use of, . 568

F.

Fever, bilious remittent, 27
epidemic duration and extent, . . 27
symtoms and progress, 28
treatment, . 29
sequelæ, and non contagion, 32
Fungus Hœmatodes, . 32
history of the case, plate, 32
remarks, . . 39
Femur, dislocation of the head of, . . 227
diagnostic symtom, 128
cases, . . 128, 129
Femoris os, fractures of, 142
Fever autumnal account of, 342
light bandages, . 343
Fever yellow, pathology and treatment, . 367
preliminary remarks, 367
remote and exciting causes, 369
exposure to cold, eating and drinking, . . 370
exercise and passions, 371
ratio symptom atum, 372
symptoms, stages, . 375
first and second, . 375
third, . . 376
postmortem appearances, 376
individual symtoms and pathology, . . 377
suppression of the secretions, . . 379, 380
anxiety and breathing, 381
sensations of the stomach, 382
black vomit, . 382
spasms and convulsions, 383
treatment, . 384
cathartics, . 389
alteratives, . 390
stimulants, . 392
Fistulæ, treatment of, 426
Fistulæ, recto-vaginal 427
Fever scarlet, belladonna, preservative power of, 431
Fungous Hœmatodes, fœtus affected with, . 431
Fœtal bones coloured with madder, . . 454
Fever, autumnal of Geo. 159
remote causes, . . 160
pathology passim, to, 179
treatment, . . 179
bleeding, . . 179
emetics, . . 180
cathartics, . 182
salivation, . 184
blisters, . . 185
opium, . . 186
sinapisms, . . 188
sulph. quina, . . 190
chronic enlargements, 192
face and forehead form of, 272
Fever, bilious œtiology of, 494

G.

Gutta serena, history of, 33
morbid appearances, 33
remarks, . 34
Glandular swellings, ointment for, . 130
Gangrena senilis pathology, 144
inflammation of the veins, 145
case, . . 146
Gall Dr. . . 151
fatal illness, case, 151
treatment, . 151, 152
postmortem examination, 152
remarks, . 153
Gorham professor, . 153
biography of, . 153, 154
funeral, . . 154
Gout and rheumatism chronic vaporised camphor for, 421
Gas oxigen, physiological effects of, . 279
Gums and alveoli fungous tumours of, . 282
Galvanic fluid developed by the nervous tissue, experiments on, . 572

H.

Hysteria, cold effusions in, 126

Hydrocele, cure for 126
Hydrocele, varieties of, 292
Herniæ, reduction of, 293
Heart, wounds of, . 295
Herniæ, incarcerated, turpentine in, . . 303
Hydrophobia, . 574
Hydrocyanic acid, treatment of poisoning with, . 576
Hœmorrhage, mode of stopping, . . 577
Hospital Surgeons at Paris, 603

I. & J.

Infants, blistering of, . 123
Jurisprudence, medical, trial for murder, . 44
plea mania a potu, . 44
physical and moral symptoms, . . 45
perpetration of the deed, 46
conduct in prison, . 47
defence, . . 47
prosecution, . 48
remarks, . . 48
mania a potu never continued into permanent insanity, 49
difference of opinion, 50
responsibility, . 50
observations, monomania, 51to7
Iodine, St. Louis hospital, 421
Jurisprudence, medical, 215
case, with remarks on feigned insanity, . 215
Intestine sloughing of twenty-six inches, . 590
Jurisprudence, medical, mania a potu, . . 598
Jefferson Medical College, 608

L.

Lithontripteur, . 117
Lungs, danger of artificial inflation, . . 125
Leontodon Taraxicum, 134
London University, . 149
organization, . 149
faculty, . . 150
Lung, hernia from tight lacing, . . 445

M.

Morbid anatomy, preserving specimens of, . 125
Mania a potu, . 135
Metallic ligature, . 148
lead, silver, . 148
gold, platinum, . 147
Measles, brief notices, 351
symptoms, . 351
treatment, . 353
Marbus cæruleus, case of, 361
Measles, cold water for, 423
Milk, suspending secretion, 442
Monstrosity, fœtal, cases, 213
Morphine therapeutic properties of, . . 268
Muscœ Volitantes, pathology, 274
Mineral substances molecules in, . . . 576
Milk sickness; facts and observations on, . 465
disease among inferior animals, . . 466
cases of Dr. Sharp, 470
editorial note, new disease, 482
age, sex, treatment, 480
Malaria, a review, . 500
production, . 502
soils and situations, . 507
soils and situations less suspected, . . 509
revolution and changes in matter, . . 527
propagation . 529
climate and season, . 539
general effects, . 542
Monster, human, . 569
Morphia, externally applied in tetanus, . 580

N.

Neuralgia facialis, . 120
Neuralgia, . . 123
Nux vomica, . . 127
diarrhea, . . 127
intestinal hæmorrhage, 127
Neuralgias, . . 138
general treatment, . 139

O.

Obstetrics, manual of 100
natural labor, . 100
preternatural labor, . 100
presentation of the feet, 101
knees, . . . 101
vertex of the head, 102
instrumental labor, 103
impaction of the head, 103
cutting instruments, 103
cæsarian operation, 103
Opium, treatment of poisoning by, . . 576
Ophthalmia gonorrhæal, treatment of, . . 583
Ophthalmia Nœontorum, 484
Obituary of Prof. Brown, 606

P.
Prussic acid, test for, 143
proto sulphate of iron, 144
People's doctors, a review, 393
of Thompson's system, 394
Robinson's lectures, 396
family medicines, . 405
treatment of diseases, 406
measles and small pox, 406
consumption, . 407
Swaim's panacea, 415
Rafinesqes' pulmel, 417
new guide to health, 418
prescriptions, . 419
silver . . 420
Palsy, partial, cured by strychnia, 434
ptyalism, remedy for, 454
people's doctors, Dr. Salmon and prescriptions, 455
Poisons, modus operandi, 305
Pregnancy stethoscope in, 594
Q.
Quinine, administration of, 276
Quinine sulphate applied to the skin in fevers, . 280
R.
Rectum, debility of, . 428
Rectum, imperforate, treatment of, . 442
case, . . 444
Rhubarb plant, . 280
Readers, . . 314
Roller-bandage, effects of, 497
cases, . . 498
S.
Scrofulous disease, by C. G. Hufeland, . 545
nature, proximate cause, 546
lymphatic system, . 546
absorption, . 547
preparation and assimution, 548
remote causes, . 549
hered. tendency, age & sex, 549
weakness & syphilis parents
unwholesome aliment, 550
unwholesome air, precocious studies, . 551
occasional exciting causes, 552
growth, season, diseases of irritation, . 552
primitive seat of the disease in the solids, . 552
proximate cause exists in the fluids as well as in the solids, . 553
Scrofulous taint in lymphatic system, . 554
desription of scrofulous diathesis, . . 555
characteristics of the dis. 556
ulcers and lymphatic swellings, . . 557
treatment, . 558
exercise, attention to cleanliness, . . 559
purgatives, antimony, 560
mercury, muriate of Baryta, . . 561
muriate of lime, bark, iron, narcotics, . 562
Silver nitrate, powers of, 569
Silver, nitrate, test for arsenic, new mode of employing, 578
Sulphate of quinine, powers, 579
Scrofula, iodine in, . 595
Society, Medical, N. Y. . 104
transactions of, . . 104
vaccination, . . 105
intemperance, . . 105
premiums, . . 106
Society, Medical, Ohio, . 107
origin of, . . 107
officers, . . 108
Small Pox, . . . 155
Spring chalibeate, . 159
Society Medical 1st dist. O. 160
transactions of, . . 160
capitation tax, . 160
resolution, . . 161
resolution against Intemp. 161
officers of, . . 161
Stomach, fatal organic dis. of, 344
dissection, . 345–6
Small pox, Pittsburgh, 447
Spina Bifida, notes of, 210
Post Mortem appearances, 211
Stranguary from cantharides 267
Sciatica, oil turpentime for 271
Silver, found in the viscera 280
Small pox, . . 283
Swaims panacea, mercury in 285
Siamese boys, account of 286
another account, . 288
Silliman's Journal, . 313
Symphysis pubis, separation of, . . 604
Subscribers and the profession generally, . 600
T.
Tenia, new mode of treat'mt. 118
No. 1, . . 119

No. 2, . . 119
Tetanus traumatic, pathol. of 129
case, . . 129
dissection, . . . 130
Tartar Emet. large doses 423–33
Tetanus, sub carb. of iron in 425
Thigh bone, fractures of treat. 445
Teeth, tartar on . 275
Taliacotian operation, 282

U.

Uterus, amp. of the neck I09
Uteri cervix, carcerous, 110
Cases, . . 111
Ulcers, varicose, . 114
treatment, . . 115
leg, . . 1!5–16
Urine, action of Colchicum 118
Urethra, structures of 124
Uteri cervix, cartilaginous structure of, . 341
Uterus, ruptured case of 206
editorial note, . 208
Uterus, infl. of mercury on 269
Menorrhagia & amenorrhea 269
abortion, . . 270
Uterine discharges, ergot in 281
Umbilicus, united in a pair of twins, 290
Ulcers and grangrene, pyroligneous acid in, . . 589

V.

Verminous disease, case of 40
treatment, . . 41
conclusion, . . 43
Vaccination, fatal effects from 156
case, . . 157
Vaccination, inflamma. from, 158
case, . . 159
Veins purulent concret. in 291
remarks, . . 292

W.

Wasp, sting of . . 126
remedy, . . . 126

